# Personal Prayer in Patients Dealing with Chronic Illness: A Review of the Research Literature

**DOI:** 10.1155/2015/927973

**Published:** 2015-02-26

**Authors:** Karin Jors, Arndt Büssing, Niels Christian Hvidt, Klaus Baumann

**Affiliations:** ^1^Caritas Science and Christian Social Work, Faculty of Theology, Freiburg University, Germany; ^2^Institute of Integrative Medicine, Faculty of Medicine, Witten/Herdecke University, Herdecke, Germany; ^3^Faculty of Health Sciences, University of Southern Denmark, Odense, Denmark

## Abstract

*Background*. Prayer is commonly used among patients for health purposes. Therefore, this review focused on three main questions: (1) why do people turn to prayer in times of illness?, (2) what are the main topics of their prayers?, and (3) how do they pray? *Method*. We undertook a systematic review of the literature by searching the databases PubMed, Medline, and PsycINFO. The following inclusion criteria were used: (1) participants in the study were patients dealing with an illness, (2) the study examined the use of private rather than intercessory prayer, and (3) the content and purpose of prayer rather than its effects were investigated. *Results*. 16 articles were included in the final review. Participants suffered from a variety of chronic diseases, mostly cancer. Five main categories for the reasons and topics of prayer were found: (1) disease-centered prayer, (2) assurance-centered prayer, (3) God-centered prayer, (4) others-centered prayer, and (5) lamentations. Among these, disease-centered prayer was most common. *Conclusions*. Although most patients with chronic diseases do pray for relief from their physical and mental suffering, the intention of their prayers is not only for healing. Rather, prayer can be a resource that allows patients to positively transform the experience of their illness.

## 1. Introduction

Personal prayer of patients has been shown to be both positively [1, 2] and negatively [3–5] correlated with their physical and mental well-being. Despite this lack of conclusive evidence on the relationship between private prayer and well-being, research has shown that prayer is commonly used for health purposes [6, 7]. Interestingly, it has been shown to be one of the most frequently used forms of “complementary and alternative medicine” (CAM) [8]. Although such classification is debated, because prayer is neither an alternative to conventional medicine nor a specific therapy in a strict medical context, the National Center for Complementary and Alternative Medicine (NCCAM), sponsored by the National Institutes of Health (NIH), considers prayer as one of many types of complementary and alternative endeavors towards improved health [9, 10]. A trial from 2004 showed that 62% of random (healthy and sick) US-Americans had used some kind of CAM, including prayer, over the last twelve months. Three forms of prayer for health were in the top four: prayer for oneself (43.0%), the prayers of others for oneself (24.4%), and participation in a prayer group (9.6%) [11]. Another trial published in 2005 with 493 patients from a Texas hospital showed that 79.2% of patients prayed for their own health and 71.9% said others prayed for them [12].

When people are confronted with an illness, particularly chronic illness, they may tend to engage in prayer and other religious practices more frequently [13]. Prayer is used by people of all theistic faiths and even by those who do not belong to a particular religious tradition [14]. In this sense, prayer is not only a basic dimension of theistic religions [15, 16] but also an essential ingredient of personal spirituality which has been considered “not as an intense form of other-worldliness remote from the common ways and incompatible with the common life, but rather as the heart of all real religion and therefore of vital concern to ordinary men and women” [17, pages 7-8]. For this reason, it is particularly interesting to examine the role of prayer among the sick.

Prayer can be defined in various ways and differs according to individual beliefs and religious traditions. For our purposes, we distinguish private prayer from personal meditation as frequently and popularly understood. Meditation is often framed as a mental exercise or state of being involving reflection/contemplation or mindfulness and is not necessarily directed toward a higher being. Prayer, on the other hand, as for example, Damascene (about 650–754 AD) put it, involves “the raising up of one's mind to God” [18]. For the purpose of this current investigation, we will define prayer as the conscious act or attempt of opening oneself or activating a relationship to a higher being (i.e., God). External attributions and so-called implicit, unconscious prayers are not included notwithstanding their theological and existential relevance [19]. Consequently, meditation can be included if understood and practiced as a conscious act of connecting to a higher being. Meditation is excluded if explicitly practiced as a mental exercise without being directed toward a higher being. In addition, one can also distinguish among different forms of prayer as defined here. In their investigations on prayer and well-being, Paloma and Pendleton identified four main types of prayer: (1) petitionary prayer, that is, specific requests for (a) oneself or (b) others; (2) colloquial prayer, that is, a conversational style of prayer in which people may ask for personal guidance, forgiveness, or general blessings; (3) ritual prayer, that is, memorized prayers or prayers from books; and (4) meditative prayer, that is, prayer involving reflection upon, and adoration of the divine. According to their results, these different types of prayer also predicted different levels of well-being; whereas ritual prayer was related to greater depression and tension, colloquial prayer was associated with greater happiness [20]. In a more recent study, Whittington and Scher made similar observations. Whereas prayers of adoration, thanksgiving, and reception were positively associated with well-being, prayers of supplication, confession, and obligation were either not at all or negatively correlated with well-being. The authors hypothesized that prayers more focused on God rather than on oneself have a more positive effect on well-being [21]. Ladd and Spilka examined the cognitive aspects of prayer and differentiated between three categories of prayer: (1) “inward prayers,” that is, prayers of self-examination or for personal formation, (2) “outward prayers,” that is, prayers involving relationships with others or external needs, and (3) “upward prayers,” that is, prayers focused on the relationship with the divine [22]. Of course, and as yet worth mentioning, these categories are not mutually exclusive. Rather, internal self-examination, for example, may lead one to recognize and pray for certain external needs. In general, most studies of prayer have focused on the active role of the person praying. However, Poloma and Lee point out that prayer involves a relationship with the divine, and thus receptive prayer, that is, openness to experiencing the love, presence, or guidance of God, should not be overlooked [23].

Prior research, such as that of Paloma and Pendleton and Ladd and Spilka, has usually focused on different types of prayer among the general population. Furthermore, past reviews of prayer in patients have focused on the effects of prayer on well-being rather than the content, motivation, and manner of prayer [24]. As Masters and Spielmans have put it, “the research is also quite limited in scope and lacking in depth, leaving open many lines of research to be studied with a variety of methods” [14, page 335].

Actually, few investigations have been conducted to examine how and why people pray particularly during times of illness. We assume that prayers of the sick differ in focus and quality from those of the general population. For this reason, we set out to conduct a review of studies that have examined how and for what intentions people pray when struck with illness. We asked three main research questions: (1) why do people turn to prayer in times of illness?, (2) what are the main topics/intentions of their prayers?, and (3) how do they pray?

## 2. Methods

In September 2013 and April 2014, we searched the databases PubMed, Medline, PsycINFO for empirical studies focusing on prayer and illness. The following English language search terms were used: “prayer∗cancer,” “prayer∗chronic,” “prayer∗disease,” “prayer∗illness,” “prayer∗pain,” and “prayer∗sickness.” We did not include the search terms “meditation” and “mindfulness” because we aimed at focusing on explicitly “religious” practices of prayer directed toward God or some transcendent being. To increase the chances of finding all relevant publications regarding the function and topics of prayer among patients dealing with an illness, there were no limitations in the initial search in terms of language, year, status, or design. In addition, we nonsystematically asked experts to provide any potentially relevant articles that may not have been found in our initial search. However, this did not result in any additional articles.

The titles and abstracts of all potentially eligible articles were screened for relevance by two independent reviewers (Karin Jors and Arndt Büssing). If an article did not have an abstract, it was automatically excluded due to scientific publishing standards. Furthermore, articles were excluded if they did not report the results of an empirical research study but rather provided, for example, case reports or theoretical reflections. After screening titles, keywords, and abstracts, all potentially relevant full-text articles were retrieved. The full text of potentially relevant articles was read thoroughly by two independent reviewers and assessed for final inclusion based on the following criteria for exclusion:

1. patients were not the main subjects;
2. intercessory prayer* for* patients rather than personal prayer was investigated;
3. prayer was only briefly mentioned, for example, in regard to complementary and alternative medicine;
4. effects rather than content/purpose/manner of prayer were investigated.

Any disagreements regarding what articles should be included in the review were resolved by discussion between both reviewers. If necessary, a third reviewer (Klaus Baumann) was consulted to reach consensus.

### 2.1. Search Results

**Figure 1:**
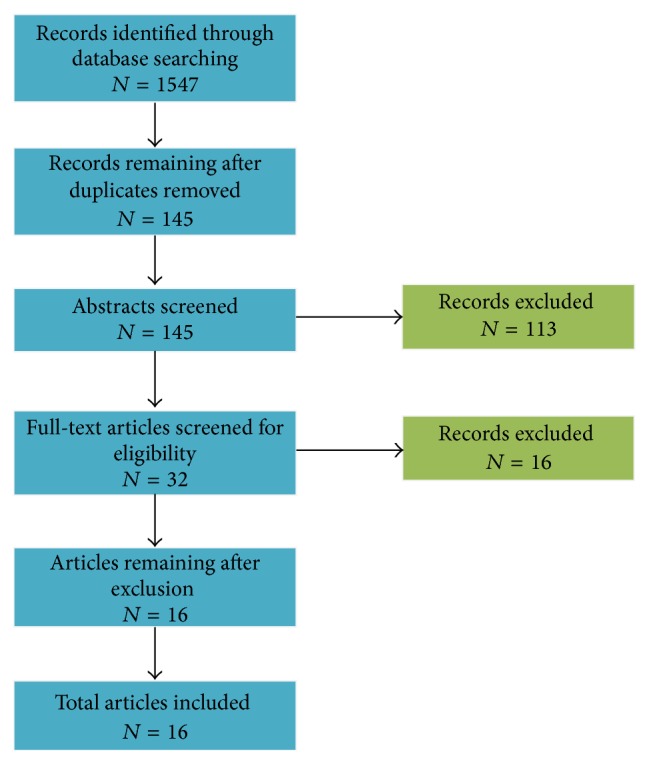
Flow diagram of studies found through database search.

Using the aforementioned search terms, we found 145 potentially relevant articles in the searched databases, after excluding duplicates. A total of 113 articles were excluded (Figure 1), mostly because they did not investigate the role of personal prayer from a patient's perspective but rather from the perspective of family members or health care professionals. After screening the abstracts of all articles, the full text of 32 empirical research studies was reviewed. Of these, 16 studies were excluded because they focused on the effects of prayer and/or did not provide sufficient information on the topics, reasons, and manner of prayer. For example, some studies discussed different forms or types of prayer that had previously been discussed in the research literature but the study itself did not investigate to what extent these forms of prayer were practiced by patients. Thus, 16 articles were reviewed in total (Figure 1).

### 2.2. Data Extraction and Analysis

After identifying articles for inclusion in the review, data from each study was extracted on the following topics: general study design (qualitative, quantitative, etc.), main study question, number of participants, patient characteristics (mean age, gender distribution, ethnicity, and religious affiliation), patient diagnosis, research instruments used, and content, reason, and manner of prayer. For the purpose of data analysis, all relevant data was entered into an Excel table. Information regarding the topics, reason, and manner of prayer was copied and pasted word for word into the table.

Data on how, why, and what patients pray was subsequently submitted to content analysis. A group of three reviewers (Karin Jors, Arndt Büssing, and Klaus Baumann) began by screening all data to gain an overall impression of the material. Next, common words and phrases were identified and grouped into thematic categories. Disagreements regarding the appropriate category were discussed to reach consensus.

### 2.3. Description of Studies

As shown in Table 1, among the selected studies the selected studies, 10 had a qualitative design (Taylor 1999 and Taylor 2002 report about the same study and sample from different perspectives), 3 a quantitative design, and 3 used mixed-methods. Semistructured interviews were the most common form of gathering data; however, one study used a focus group discussion and others implemented previously tested questionnaires (e.g., Poloma and Pendleton's Prayer Scale).

The number of participants in the included studies ranged from 10 to 360 (total 1,545, counting the Taylor 1999 and 2002 sample only once). In 9 studies, patients had been diagnosed with cancer; the remaining 7 studies investigated patients with various chronic diseases such as sickle cell disease, heart problems, HIV, and Hepatitis C. Two studies included children/adolescents <18 years in their sample. In over half of the 16 included studies, the average age was above 50 years. Overall, there were more female (*n* = 862) than male (*n* = 683) participants in the studies; 5 studies included only female participants.

Although most study populations consisted of a variety of ethnicities, the majority of participants were Caucasian, followed by African American. Most studies were also conducted in the United States. Five studies did not include any information regarding the religious background of the participants; of the remaining studies, the majority consisted of participants from Christian denominations. Only one Iranian study (Rezaei et al., 2008) specifically focused on participants with Muslim beliefs [25].

## 3. Results 

### 3.1. What and Why Patients Pray

Our findings revealed that the topics of patients' prayers often correlate with the reasons why they pray. For example, improvement in health can be both: the motivation for prayer and the content of prayer. For this reason, we have chosen to discuss these two aspects together. As shown in Table 2, we identified 14 initial categories which were condensed into five central categories of prayer: (1) disease-centered prayer, (2) assurance-centered prayer, (3) God-centered prayer, (4) others-centered prayer, and (5) lamentations. Characteristic quotes for each category can be found in Table 3. 

*(1) Disease-Centered Prayer*. Disease-centered prayers were directly related to patients' illness and were by far the most commonly mentioned prayers. Table 2 shows that prayers in this category were for (1) improvement in physical health or state of min, (2) disease management and decision-making, and (3) positive contributions to their experience of disease. As depicted in Table 2, prayers for improvement in physical health and state of mind were mentioned in all 16 studies. Eleven studies reported that patients prayed for guidance regarding the management of their disease, and seven studies found that patients used prayer to find meaning in their experience of the disease. 

*(2) Assurance-Centered Prayer*. The second most commonly mentioned prayers were assurance-centered prayers (Table 2), which provide patients with the confidence and comfort that their God does and will continue to take care of them even in the face of disease or wrongdoings. Subcategories of assurance-centered prayers included (1) protection, (2) strength/hope, (3) trust, (4) gratitude, and (5) forgiveness and guilt. Prayers of protection were often prayed before surgery or to keep the disease from worsening. Despite their disease, patients continued to thank God for their blessings, which reassured them of God's goodness. Some patients found that prayer challenged or even forced them to confront mistakes they had made, which prompted them to also ask for forgiveness. 

*(3) God-Centered Prayer*. God-centered prayers are those which are specifically focused on the relationship between God and the patient. In these prayers, the needs of the patient are secondary to God's greatness. We identified the following subcategories of God-centered prayers (Table 2): (1) relationship with God (e.g., guidance and conversation with God), (2) worship and adoration, and (3) reflecting on and experiencing God's presence. Prayer focused on reflection and the experience of God's presence can be equated with what Poloma and Lee described as “receptive prayer” [23]. 

*(4) Others-Centered Prayer*. Six studies mentioned that patients prayed for others (Table 2). These prayers included prayers not only for family and friends but also for their physicians. Taylor et al. reported that in fact some patients prayed only for others and perceived prayers for themselves as selfish [26]. 

*(5) Lamentations*. In the studies reviewed here, the least commonly reported topic of prayer was lamentation. This category included prayers of (1) fear and complaint and (2) doubt. Taylor was the only author to discuss patients' doubt that God answered their prayers or could heal their disease [26, 27]. Prayers of fear and complaint addressed in part the question of theodicy, that is, why their good and almighty God allows bad things to happen [26, 27]. In the study by Walton and Sullivan, some patients also expressed their fear to God regarding operations [28].

### 3.2. How Patients Pray

Regarding the question of how patients pray, not all studies provided information. Of those that did address this issue, a great variety was found. In terms of the form of prayer, several patients preferred informal, conversational prayer [25–32], while some also relied on formal or memorized prayers such as the rosary or the Our Father among Christians [26, 29, 30, 32–34] or the “seven tools” utilized by Muslims [25]. Various assistive techniques for praying were mentioned including the use of particular postures such as kneeling or bowing [27, 33], reading religious material [27], journaling [27, 35], visualization [35], breathing [27], and the lighting of candles [32].

The time and place of prayer were often considered by patients to be very flexible [27, 33, 36]. For example, Harvey and Silverman wrote that “Praying for one's health did not require a specific time or place. Prayer occurred first thing in the morning, throughout the day, before going to bed, on the bus, in the doctor's office, in the dialysis clinic, in church, in their homes…” [36, page 7]. Some patients reported, however, that they prayed at particular times such as before taking medications, when pain was difficult to bear, before surgery, in the morning, and in the evening [28, 32, 35–38].

Some also preferred certain places for prayer, such as a church or a quiet room [27, 34]. In some studies, participants also reported that prayer with others (e.g., in a group or with a family member) was particularly helpful [26, 27, 32, 34, 36, 38, 39]. In one study, participants also turned to the saints for support in their prayers [34].

## 4. Discussion 

The purpose of this review was to investigate what, why, and how patients pray. Our findings reveal that even in the face of illness, prayer remains multifaceted. Five main categories for the content and purpose of prayer were identified: (1) disease-centered prayer, (2) assurance-centered prayer, (3) God-centered prayer, (4) others-centered prayer, and (5) lamentations. One should of course note that these categories are not mutually exclusive.

Not surprisingly, disease-centered prayer was most common. However, although patients often prayed for their disease to improve or their pain to be relieved, they did not always believe or even hope for a cure. As one participant noted, “I think God can take that (cancer) away in a second if He wants, (but) I don't pray for that though because I don't know if that's what He wants.” [27, page 53]. Patients may prefer to avoid asking for physical healing out of fear that God will not answer their prayer or as surrender to God's will (esp. Muslims, cf. Rezaei et al. 2008). As Dein and Pargament suggest, prayers for psychological rather than physical changes may help people to avoid cognitive dissonance and continue to believe that God can, if he wants, intervene in the world [40]. For this reason, it is not surprising that prayers regarding disease management and decision-making were nearly as common as prayers for the disease itself. Participants also prayed for positive contributions to their experience of the disease, that is, the ability to find meaning or something positive in their disease. According to Pargament, religion and its expression through prayer help people to understand and bear suffering by viewing them in a larger, spiritual context [41].

One surprising result of this review was that only very few studies mentioned prayers of lamentation, that is, complaint, fear, or doubt, although most religions know such struggles as inherent aspects of faith. This raises the question of whether researchers have turned a blind eye to these less pleasant aspects of prayer or whether they truly play a minor role for patients. One possible explanation is that patients are “just as invested in conserving/protecting the divine through their prayers as they are in conserving and protecting themselves” and are thus afraid to admit their frustration with God [40], maybe especially in the context of a clinical survey with its dynamics like social desirability and observers' effect. This may be due to “dogmatic expectations” (like in Islam; cf. Rezaei 2008) or to cultural and/or denominational (self-) expectations and differences among Christian groups. However, researchers may also have chosen to avoid addressing these issues because they might believe that such unpleasant aspects of prayer do not benefit patients' well-being and do not help patients to cope. Although desperate pleas and complaints to God may be associated with a greater level of anxiety, this aspect of prayer of patients deserves further research, whatever the physiological associations. Prayers of fear, complaint, and doubt are a central motif in the biblical psalms and are often reported today by clinical chaplains. In fact, they play an important role in spiritual traditions, as epitomized in the biblical person and experience of Job. Are doubts, despair, desolation, and complaints not part of the religious experience of patients which may find expression in religion's natural language of prayer? The open expression of doubts, complaints, and feelings of injustice in front of God may not only have a positive psychological, “cathartic” effect of “letting go” but also be part of an authentic spiritual process in “truthful relation” with God during disease.

In addition to a lack of focus on unpleasant aspects of prayer such as complaint or doubt, our review of the literature revealed further blind spots in research on prayer among those suffering from disease. Foremost, there is a comparative richness of data on patients from Christian backgrounds. Only one study in our review specifically investigated prayer among Muslim patients; none focused exclusively on Jewish or Hindu patients. Furthermore, a majority of studies investigated the use of prayer in patients suffering from cancer. Other chronic diseases received limited attention. Participants in most studies were on average over 50 years of age. It would be interesting to investigate prayer among a greater variety of populations, in regard to the disease, culture, and stage of life. After all, the aphoristic statement of a hospital chaplain and retreat director seems to reflect the deeply human reality hidden in most hospitals, that is, that the most intense spiritual retreats are not usually lived and worked through in houses of retreat but in hospital beds [32].

## 5. Clinical Relevance

At first glance, the personal prayer practices of patients may not appear relevant to health care professionals. However, we believe our findings can be useful for clinical practice because praying can be regarded as a strategy to cope and to connect with a higher source providing meaning and hope [42, 43]. Our results have shown that many patients turn to prayer for guidance regarding treatment decisions and disease management. Physicians should be aware of this possibility and may find it helpful to address the influence of patients' prayer on their treatment decisions in a short spiritual history [44].

The results of this review on how patients pray may also be useful in the clinical setting in order to provide a suitable environment for prayer. For example, health care professionals may cooperate with the clinical chaplain to provide spiritual reading material or a quiet environment in which to pray. Whether health care professionals should offer to pray with patients is debated among professionals. On the other hand, in a study by Balboni et al. (2011), many patients expressed openness to the idea of patient-practitioner prayer [45].

## Figures and Tables

**Table 1:** Overview on identified studies.

Author, year	Main question	Type of study	Diagnosis	*N* (f/m)	Mean age, range (years)	Religious background of participants	Central findings
Ai et al., 2002 [29]	How is private prayer used among middle-aged and older patients to cope with cardiac surgery?	Mixed-methods	Heart problems	246 (109/137)	62, 36–86	Protestant: 52.8% Catholic: 26.8% Orthodox: 2.0% Jewish: 3.7% Other religions: 3.7% No preference: 11.0%	88% of participants expressed a belief in the importance of prayer and used private prayer as a way to deal with surgery-related difficulties. The most common form of prayer was “conversation with God.” Prayer was positively correlated with optimism.

Ai et al., 2007 [30]	How is prayer for coping with cardiac surgery associated with patients' outcomes?	Mixed-methods	Heart problems	310 (130/180)	62, 35–89	Judeo-Christian: 87%	88% of participants expressed a belief in the importance of prayer and used private prayer as a way to deal with surgery-related difficulties. The most common form of prayer was “conversation with God.” Prayer was positively correlated with optimism.

Cotton et al., 2012 [46]	What is the content and frequency of prayer among children with SCD?	Qualitative (semistructured interviews)	Sickle cell disease	19 (11/8)	8, 5–10	Protestant: 47% Catholic: 16% None: 16% Others: 16%	A majority of the children used prayer/religion to manage their disease and prayers were mostly about getting well, not getting sick again, or getting out of the hospital.

Crane et al., 2000 [33]	What role does prayer play in HIV-infected women's decisions regarding treatment options?	Qualitative (semistructured interviews)	HIV	51 (51/0)	13–19: 10%; 20–29: 31%; 30–39: 35%; 40 and up: 24%	No information	Nearly all participants believed that prayer was an important part of HIV decision-making and that prayer allowed them to accept their disease.

Harvey and Silverman 2007 [36]	What role does spirituality play in the self-management of chronic illness in adults?	Qualitative (semistructured interviews)	Chronic illness	88 (41/47)	74	Protestant: 68.2% Catholic: 27.3% Others (1 Jewish, 1 none): 4.5%	Prayer played a vital role in the self-management of chronic disease and helped patients accept their illness. Participants asked for alleviation from their illness and used prayer to shape their experience of the disease.

Harvey and Cook 2010 [37]	What role does spirituality play in the self-management of chronic illness among adult women with chronic conditions?	Qualitative (semistructured interviews)	Chronic illness	41 (41/0)	73, 66–85	No information	Prayer was used as a method of pain management and many participants experienced relief from their pain as the result of prayer.

Klafke et al., 2014 [39]	How and why do Australian men with cancer practice complementary therapies (CTs) and how do their significant others contribute to the regular uptake of CTs?	Qualitative (semistructured interviews)	Cancer	26 (0/26)	68, 61–75	No information	Participants used meaning-based coping, that is, prayer, to cope with physical, emotional, and spiritual issues related to their disease.

Lagman et al., 2014 [34]	What meaning does spirituality and religion have for Filipina immigrants with a breast cancer diagnosis?	Qualitative (semistructured interviews)	Breast cancer	10 (10/0)	54	Catholic: 100%	Prayer was the most common religious practice for coping with the disease. Prayer helped participants to find strength and see their illness in a positive light.

Levine et al., 2009 [35]	How does the use of prayer differ between breast cancer survivors from different ethnic groups and how is it related to mood and quality of life?	Mixed-methods	Breast cancer	175 (175/0)	58, 31–74	Catholic: 25% Protestant: 33% Jewish: 4% Buddhist: 4% Others: 4% None/nonpracticing: 18%	Women who prayed were able to find more positive contributions from their breast cancer experience. Most women prayed for healing but also offered thanks and asked for guidance, strength, comfort, and protection before surgery or for others.

Meraviglia 2002 [31]	How can an instrument for assessing prayer activities, experiences, and attitudes of people with cancer be adapted?	Cross-sectional survey	Cancer	32 (24/8)	56, 31–74	Christian: 100%	Prayers helped patients adjust to their diagnosis and provided them with guidance regarding treatment decisions. As physical functioning decreased, the use of prayer increased.

Rezaei et al., 2008 [25]	How does prayer impact Iranian cancer patients undergoing chemotherapy?	Cross-sectional survey	Cancer	360 (171/189)	20–39 years: 46.1%; 40–59 years: 36.9%; 60-61 years: 17%	Muslim: 100%	Participants most often prayed for forgiveness and for guidance in decision-making. Individual characteristics (e.g. age, education, and sex) had an important effect on prayer.

Richmond et al., 2010 [38]	Why do adults with CHC use mind-body medicine?	Mixed-methods	Hepatitis C	133 (70/63)	52	No information	88% of participants reported praying for their health as a type of mind-body medicine and felt that prayer provided emotional support and improved the effectiveness of conventional medical treatments.

Smith et al., 2012 [32]	How do patients with advanced cancer pray and how is prayer used to cope with cancer?	Qualitative (focus group)	Lung/ovarian cancer	13 (13/0)	Late 30 s–early 80 s	No information	Prayer was used to find one's own way, find renewed appreciation for life, gain strength and courage, and strengthen their spiritual connection. Participants most often used conversational prayer, petitionary prayer, ritual prayer, and thanksgiving prayer.

Taylor et al., 1999 [26]	What spiritual conflicts are experienced by persons with cancer?	Qualitative (semistructured interviews)	Cancer	30 (16/14)	58, 19–77	Christian (various denominations), Jewish	Many participants struggled to pray for certain things, such as a cure. Also, participants questioned the meaning of having cancer, the nature of God, why God does not always answer prayers.

Taylor and Outlaw 2002 [27]	Why, when, and how do person with cancer pray and what outcomes do they expect?	Qualitative (semistructured interviews)	Cancer	30 (16/14)	58, 19–77	Christian (various denominations), Jewish	Participants prayed to ease the physical, emotional, and spiritual distress of their disease. In particular, patients often prayed for healing, guidance regarding treatment, and help getting through day to day life.

Walton and Sullivan 2004 [28]	What meaning does spirituality have for men with prostate cancer and how does it influence their treatment?	Qualitative (semistructured interviews)	Prostate cancer	11 (0/11)	54–71	Christian: 100%	Prayer was an important aspect of coping with cancer and provided them with hope and inner strength. Participants shared their fears with God and asked for guidance regarding treatment decisions.

**Table 2:** Frequency of the topics of/reasons for prayer.  ** **

Main categories	Subcategories	Ai et al., 2002 [29] *N* = 246	Ai et al., 2007 [30] *N* = 310	Cotton et al., 2012 [46] *N* = 19	Crane et al., 2000 [33] *N* = 51	Harvey and Silverman 2007 [36] *N* = 88	Harvey and Cook 2010 [37] *N* = 41	Klafke et al., 2014 [39] *N* = 26	Lagman et al., 2014 [34] *N* = 10	Levine et al., 2009 [35] *N* = 175	Meraviglia 2002 [31] *N* = 32	Rezaei et al., 2008 [25] *N* = 360	Richmond et al., 2010 [38] *N* = 133	Smith et al., 2012 [32] *N* = 13	Taylor et al., 1999 [26] *N* = 30	Taylor and Outlaw 2002 [27] *N* = 30	Walton and Sullivan 2004 [28] *N* = 11
Disease-centered prayers	Improvement in health or state of mind	**x**	**x**	**x**	**x**	**x**	**x**	**x**	**x**	**x**	**x**	**x**	**x**	**x**	**x**	**x**	**x**
	Disease management			**x**	**x**	**x**	**x**	**x**	**x**				**x**	**x**	**x**	**x**	**x**
	Positive contributions				**x**				**x**	**x**				**x**	**x**	**x**	**x**

Assurance-centered prayers	Protection		**x**	**x**					**x**	**x**		**x**	**x**			**x**	
	Strength/hope				**x**		**x**		**x**	**x**			**x**	**x**	**x**		**x**
	Trust				**x**		**x**		**x**		**x**		**x**			**x**	**x**
	Gratitude	**x**	**x**							**x**	**x**	**x**	**x**	**x**		**x**	**x**
	Guilt and forgiveness									**x**	**x**	**x**			**x**	**x**	

God-centered prayers	Worship/adoration										**x**			**x**			**x**
	Reflecting on and experiencing God's presence	**x**	**x**			**x**					**x**	**x**			**x**		**x**
	Relationship with God	**x**	**x**		**x**						**x**	**x**		**x**	**x**	**x**	**x**

Other-centered prayers	For others		**x**			**x**				**x**				**x**		**x**	**x**

Lamentations	Complaint and fear														**x**	**x**	**x**
	Doubt														**x**	**x**	

**Table 3:** Example quotes for each category.

Category	Example quote	Study
Improvement in health and/or state of mind	“When I get pains and soreness—soreness and pain or aches or whatever, I pray to God He relieve it; cast it out of my body…He's the only one that can heal it.”	Harvey and Silverman 2007 (p. 211) [36]

Disease management and decision-making	“My doctor wanted me to go on medication and I told [them] that I have to think about it, but I just pray about it…I was scared, but I prayed and asked God for wisdom and knowledge.”	Crane et al., 2000 (p. 536) [33]

Positive contributions for the experience of the disease	“When I came to the hospital, I did a little bit of praying, and then after that, I started believing that I needed to live each day at a time and take advantage of this time I have, you know? I don't feel like putting things off any longer. I realized that I needed to get things, certain things, accomplished and enjoy the day. That's what spirituality meant to me so far.”	Walton and Sullivan 2004 (p. 143) [28]

Relationship with God (e.g., guidance and conversation with God)	“I realize that God is around me and in me, and He's my friend, because I talk to Him like we're really pals, although I know He's God, He's a pal-God. He's like the closest pal I know that I can talk to about the hardest things that I have to think about.”	Walton and Sullivan 2004 (p. 137) [28]

Worship and adoration	“And then you know just do your praising. I praise you and I thank you and I praise you and every time thank you Jesus. I praise you Jesus. That's all. It's very simple.”	Levine et al., 2009 (p. 9) [35]

God's presence	“I use both prayer and meditation. It's nice to pray about things, and it's also nice to sit there and be very quiet and relax and listen for answers.”	Walton and Sullivan 2004 (p. 138) [28]

Prayers for others	“I know when you have an illness, you have the focus of just praying, not only for yourself, but for your family and the people you love and the people that need you.”	Smith et al., 2012, (p. 313) [32]

Protection	“The prayer protection. You know, “The love of God surrounds me, the love of God unfolds me, the power of God protects me, and the presence of God watches over me. For wherever I am God is and always shall be.” So I said that prayer—I said it before I went into surgery then and I said it in the—in the surgery room for breast cancer.”	Levine et al., 2009 (p. 9) [35]

Strength and hope	“[I pray] I need strength today. Today is a hard day. Get me to my next visit. It's kind of like bribery to me. I don't ever…If you [God] take something away from me, then you have got to give me strength to deal with it.”	Taylor et al., 1999 (p. 390) [26]

Gratitude	“I just pray, I pray every morning. I pray every morning, and thank Him for …either your cup is half full or your cup is half empty. When I get up, I thank God because I have another day.”	Smith et al., 2012 (p. 313) [32]

Trust	“I think when you pray about something, you can only take it to God one time. So when I pray, I say I'm leaving it in your hands.”	Harvey and Silverman 2007 (p. 214) [36]

Forgiveness and guilt	“If in prayer you recognize you've done something wrong, it doesn't make you feel good.”	Taylor and Outlaw 2002 (p. 55) [27]

Fear and complaint	“There are times when I say “why me?” because I know a lot of people who—to put it bluntly—who deserve something hard to happen and not a thing seems to happen to them. But I don't have no control over it and it gives me some comfort to talk to Whoever it is upstairs.”	Taylor et al., 1999 (p. 390) [26]

Doubt	“It's very hard for me to believe that there's One Person that hears everybody's Prayers… I just leave my illness to God; but He may not have all power over it.”	Taylor et al., 1999 (p. 390) [26]

## Limitations

This review provides a structured picture of what research has grasped about the personal or private prayer of patients suffering from chronic physical disease. It does not offer clues to some kind of disease-specific prayer activities or empirical data on differences between the prayer of patients and prayer of general population. This may be one possible direction for future research along with investigating prayer among patients with acute diseases. Although an attempt was made to include all relevant studies, it is possible that some may not have been found. In addition, this review does not provide a thorough criticism of the methods and strategies of the papers included. Furthermore, it was not our aim to conduct a meta-analysis, and therefore, this review has not included statistical analyses on the samples from the included 16 studies. Nevertheless, this review provides a detailed picture of the current research and indicates a need and possible directions for future research.

## References

[1] Maltby J., Lewis C. A., Day L. (2008). Prayer and subjective well-being: the application of a cognitive-behavioural framework. *Mental Health, Religion and Culture*.

[2] Francis L. J., Kaldor P. (2002). The relationship between psychological well-being and Christian faith and practice in an Australian population sample. *Journal for the Scientific Study of Religion*.

[3] Koenig H. G. (1998). Religious attitudes and practices of hospitalized medically ill older adults. *International Journal of Geriatric Psychiatry*.

[4] Andersson G. (2008). Chronic pain and praying to a higher power: useful or useless?. *Journal of Religion & Health*.

[5] Rippentrop A. E., Altmaier E. M., Chen J. J., Found E. M., Keffala V. J. (2005). The relationship between religion/spirituality and physical health, mental health, and pain in a chronic pain population. *Pain*.

[6] Ai A. L., Bolling S. F., Peterson C. (2000). The use of prayer by coronary artery bypass patients. *International Journal for the Psychology of Religion*.

[7] McCaffrey A. M., Eisenberg D. M., Legedza A. T. R., Davis R. B., Phillips R. S. (2004). Prayer for health concerns: results of a national survey on prevalence and patterns of use. *Archives of Internal Medicine*.

[8] Yates J. S., Mustian K. M., Morrow G. R. (2005). Prevalence of complementary and alternative medicine use in cancer patients during treatment. *Supportive Care in Cancer*.

[9] National Center for Complementary and Alternative Medicine (2008). *Complementary, Alternative, or Integrative Health: What's in a Name?*.

[10] Verhoef M. J., Balneaves L. G., Boon H. S., Vroegindewey A. (2005). Reasons for and characteristics associated with complementary and alternative medicine use among adult cancer patients: a systematic review. *Integrative Cancer Therapies*.

[11] Barnes P. M., Powell-Griner E., McFann K., Nahin R. L. (2004). Complementary and alternative medicine use among adults: United States, 2002. *Advanced Data*.

[12] Richardson M. A., Sanders T., Palmer J. L., Greisinger A., Singletary S. E. (2000). Complementary/alternative medicine use in a comprehensive cancer center and the implications for oncology. *Journal of Clinical Oncology*.

[13] Ambs A. H., Miller M. F., Smith A. W., Goldstein M. S., Hsiao A.-F., Ballard-Barbash R. (2007). Religious and spiritual practices and identification among individuals living with cancer and other chronic disease. *Journal of the Society for Integrative Oncology*.

[14] Masters K. S., Spielmans G. I. (2007). Prayer and health: review, meta-analysis, and research agenda. *Journal of Behavioral Medicine*.

[15] Glock C. Y., Stark R. (1971). *Religion and Society in Tension*.

[16] Smart N. (1996). *Dimensions of the Sacred. An Anatomy of the World's Beliefs*.

[17] Underhill E. (1993). *The Spiritual Life*.

[18] Damascene J. De fide orthodoxa.

[19] Frankl V. (1977). *The Unconscious God*.

[20] Paloma M. P., Pendleton B. F. (1991). The effects of prayer and prayer experiences on measures of general well-being. *Journal of Psychology and Theology*.

[21] Whittington B. L., Scher S. J. (2010). Prayer and subjective well-being: an examination of six different types of prayer. *International Journal for the Psychology of Religion*.

[22] Ladd K. L., Spilka B. (2002). Inward, outward, and upward: cognitive aspects of prayer. *Journal for the Scientific Study of Religion*.

[23] Poloma M. M., Lee M. T. (2011). From prayer activities to receptive prayer: godly love and the knowledge that surpasses understanding. *Journal of Psychology and Theology*.

[24] Hollywell C., Walker J. (2009). Private prayer as a suitable intervention for hospitalised patients: a critical review of the literature. *Journal of Clinical Nursing*.

[25] Rezaei M., Adib-Hajbaghery M., Seyedfatemi N., Hoseini F. (2008). Prayer in Iranian cancer patients undergoing chemotherapy. *Complementary Therapies in Clinical Practice*.

[26] Taylor E. J., Outlaw F. H., Bernardo T. R., Roy A. (1999). Spiritual conflicts associated with praying about cancer. *Psycho-Oncology*.

[27] Taylor E. J., Outlaw F. H. (2002). Use of prayer among persons with cancer. *Holistic Nursing Practice*.

[28] Walton J., Sullivan N. (2004). Men of prayer: spirituality of men with prostate cancer: a grounded theory study. *Journal of Holistic Nursing*.

[29] Ai A. L., Peterson C., Bolling S. F., Koenig H. (2002). Private prayer and optimism in middle-aged and older patients awaiting cardiac surgery. *Gerontologist*.

[30] Ai A. L., Peterson C., Tice T. N., Rodgers W., Bolling S. F. (2007). The influence of prayer coping on mental health among cardiac surgery patients: the role of optimism and acute distress. *Journal of Health Psychology*.

[31] Meraviglia M. G. (2002). Prayer in people with cancer. *Cancer Nursing*.

[32] Smith A. R., De Santo-Madeya S., Pérez J. E. (2012). How women with advanced cancer pray: a report from two focus groups. *Oncology Nursing Forum*.

[33] Crane J. R., Perlman S., Meredith K. L. (2000). Women with HIV: conflicts and synergy of prayer within the realm of medical care. *AIDS Education and Prevention*.

[34] Lagman R. A., Yoo G. J., Levine E. G., Donnell K. A., Lim H. R. (2014). ‘Leaving it to God’ religion and spirituality among Filipina immigrant breast cancer survivors. *Journal of Religion and Health*.

[35] Levine E. G., Aviv C., Yoo G., Ewing C., Au A. (2009). The benefits of prayer on mood and well-being of breast cancer survivors. *Supportive Care in Cancer*.

[36] Harvey I. S., Silverman M. (2007). The role of spirituality in the self-management of chronic illness among older African and Whites. *Journal of Cross-Cultural Gerontology*.

[37] Harvey I. S., Cook L. (2010). Exploring the role of spirituality in self-management practices among older african-american and non-Hispanic white women with chronic conditions. *Chronic Illness*.

[38] Richmond J. A., Bailey D. E., McHutchison J. G., Muir A. J. (2010). The use of mind-body medicine and prayer among adult patients with chronic hepatitis C. *Gastroenterology Nursing*.

[39] Klafke N., Eliott J. A., Olver I. N., Wittert G. A. (2014). Australian men with cancer practice complementary therapies (CTs) as a coping strategy. *Psycho-Oncology*.

[40] Dein S., Pargament K. (2012). On not praying for the return of an amputated limb: conserving a relationship with God as the primary function of prayer. *Bulletin of the Menninger Clinic*.

[41] Pargament K. (1997). *The Psychology of Religion and Coping: Theory, Research, Practice*.

[42] Büssing A., Matthiessen P., Ostermann T. (2012). Engagement of patients with chronic diseases in spiritual and secular forms of practice: results iwth the shortened SpREUK-P SF17 questinonaire. *Integrative Medicine: A Clinician's Journal*.

[43] Büssing A., Wirth A.-G., Humbroich K. (2013). Faith as a resource in patients with multiple sclerosis is associated with a positive interpretation of illness and experience of gratitude/awe. *Evidence-Based Complementary and Alternative Medicine*.

[44] Puchalski C. M. (2010). Formal and informal spiritual assessment. *Asian Pacific Journal of Cancer Prevention*.

[45] Balboni M. J., Babar A., Dillinger J. (2011). “It depends”: viewpoints of patients, physicians, and nurses on patient-practitioner prayer in the setting of advanced cancer. *Journal of Pain and Symptom Management*.

[46] Cotton S., Grossoehme D., McGrady M. E. (2012). Religious coping and the use of prayer in children with sickle cell disease. *Pediatric Blood & Cancer*.

